# Cone parameters in different vision levels from the adaptive optics imaging

**DOI:** 10.1097/MD.0000000000025618

**Published:** 2021-04-23

**Authors:** Huanhuan Cheng, Kenneth J. Ciuffreda, Huilu Jiang, Kun Zhou, Sigeng Lin, Jingwei Zheng, Xinping Yu, Balamurali Vasudevan, Yuanbo Liang

**Affiliations:** a Department of Ophthalmology, the First People's Hospital of Wuhu City, Wuhu; b The Eye Hospital, School of Ophthalmology and Optometry, Wenzhou Medical University, Wenzhou, China; c Department of Biological and Vision Sciences, State University of New York College of Optometry; d National Clinical Research Center for Ocular Disease, Wenzhou, China; e The First Affiliated Hospital of Northwestern University,Xian,China; f College of Optometry, Midwestern University, Glendale, Arizona.

**Keywords:** adaptive optics, cone density, cone spacing, parafovea, visual resolution

## Abstract

To investigate the relationship between visual resolution and cone parameters in eyes with different levels of best corrected visual acuity (BCVA).

Seventeen eyes of 10 volunteers with BCVA of 20/12.5 or better (group 1) and 16 eyes of 10 volunteers with BCVA of 20/16 (group 2) were investigated in the study. Images of the cone photoreceptors at 1.5^°^ from the fovea were obtained using an adaptive optics (AO) retinal camera. The BCVA was obtained following a subjective refraction using a standardized logMAR visual acuity chart.

The mean cone density (29,570.96 ± 2489.94 cells/mm^2^) at 1.5° from the fovea in group 1 (BCVA ≥ 20/12.5, n = 17) was significantly greater (*P* < .001) than that (22,963.59 ± 2987.92 cells/mm^2^) in group 2 (BCVA = 20/16, n = 16). The cone spacing at 1.5^°^ from the fovea in group 1 was 6.45 ± 0.28 μm (mean ± SD), which was significantly smaller (*P < *.001) than 7.36 ± 0.50 μm (mean ± SD) in group 2. In the stepwise regression analysis, greater angular cone density (odds ratio [OR], 4.48; *P* = .005) and smaller angular cone spacing (OR, 0.60; *P* = .007) at 1.5^°^ from the fovea were significantly associated with the better BCVA.

The greater cone density and smaller cone spacing at the parafovea were found in eyes with BCVA of 20/12.5 or better, as compared to that in eyes with BCVA of 20/16. Knowledge of cone distribution for different BCVA levels may be beneficial for different clinical conditions.

## 1 Introduction

Photoreceptors are the first to process and transmit visual information to higher levels in the brain. In previous studies, understanding of the distribution of cone photoreceptors depended on histological specimens.^[1–5]^ However, with the emergence of the high resolution adaptive optics (AO) retinal camera, imaging of the individual human retinal cones is possible.^[6]^ In addition, intrasession and intersession repeatability of the AO fundus camera has been found to be excellent for the assessment of cone density and cone spacing.^[7]^

Several related studies have been performed. While images of the cones were estimated qualitatively in some retinal diseases,^[8–11]^ cone density of healthy subjects has been analyzed quantitatively using the AO retinal system.^[7,12,13]^ In addition, the effect of axial length (AL) has also been investigated.^[12,14]^Furthermore, other studies have explored and assessed the average cone parameters at different retinal eccentricities. ^[15–17]^ For example, Rossi et al^[18]^ explored the relationship between visual resolution and cone parameters in the retina. They found that only cone spacing was significantly associated with visual resolution at the fovea. However, their study focused on the relationship between visual resolution and cone parameters at fovea and outside of fovea and there have been very limited investigations specifically to address the relationship between best corrected visual acuity (BCVA) level and retinal cone parameters in healthy subjects. This is important, as the distribution of cone photoreceptors may play an important role on the BCVA range of normal visual acuities, and data from healthy subjects with different BCVA may help build the data of normal eyes with AO fundus camera.

Here, this is a cross-sectional study with healthy peoples with different levels of BCVA. Due to the limitations of the AO retinal camera (rtx1) to resolve the foveal cones sufficiently, the aim of the present study was to compare retinal images for different cone parameters at the parafovea in 2 different normal visual acuity groups based on different BCVA levels, and furthermore to evaluate whether visual resolution was associated with density and spacing of cone photoreceptors at the parafovea, even in individuals with the very high visual acuity levels.

## 2 Materials and methods

### 2.1 Subjects

**Figure 1:**
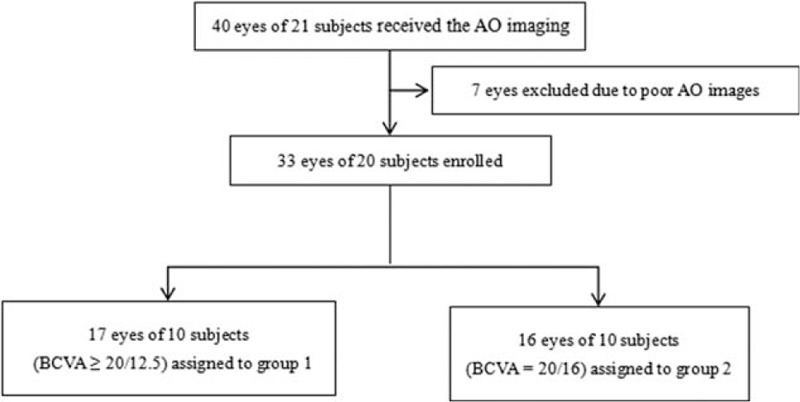
The flowchart was used to show the research methodology.

The study was performed in accordance with the tenets of the Declaration of Helsinki and approved by the ethics committee of the Wenzhou Medical University. After explaining the procedure and possible study consequences, written informed consent was obtained from all subjects recruited (Fig. 1).

Inclusion criteria were:

1. monocular best corrected visual acuity (BCVA) >20/20;
2. the eye with the better BCVA was selected if subjects had unequal visual acuity between the eye of 2 lines or greater;
3. spherical equivalent (SE) range (−6.00–0 D);
4. age range between 18 and 30 years.

A total of 40 eyes from 21 subjects were enrolled with monocular BCVA levels of 20/20 or better.

The exclusion criteria were:

1. history of ocular surgical treatment or eye trauma;
2. history of ocular disease or cataract;
3. nystagmus, unsteady fixation, or abnormal head movement;
4. retinal images of low quality that could not be analyzed by the software (AO detect v0.1).

In addition, all subjects underwent a comprehensive slit-lamp examination, a computerized subjective refraction (RT-5100, NIDEK, Tokyo, Japan), and distance BCVA testing using the logMar chart. The optical biometry parameters assessed included AL, cornea thickness, anterior chamber depth (AD), lens thickness, corneal curvature, and corneal astigmatism, all as measured by the Lenstar LS 900 biometer (Haag Streit, Bern, Switzerland). Cone parameters were obtained using the AO fundus camera (rtx1, Imagine Eyes, Orsay, France). Seven eyes were excluded from the study due to an inability to analyze the retinal Images using the software (AO detect v0.1). Hence, a total of 33 eyes of 20 subjects were assessed in the study. These subjects were subdivided based on BCVA: group 1 included 17 eyes of 10 subjects (BCVA ≥ 20/12.5), and group 2 included 16 eyes of 10 subjects (BCVA = 20/16).

### 2.2 Apparatus

The adaptive optics fundus camera was used to acquire high-resolution fundus images. Briefly, the AO retinal camera consisted of 3 main components: a low-noise charge-coupled device (CCD) camera, a HASO 32-eye Shack–Hartmann wave-front sensor, and a mirao 52-e electromagnetic deformable mirror. The rtx1 uses en-face reflectance imaging with flashed, non-coherent flood illumination. The camera has a pixel pitch of 1.6 μm on the fundus, and 850 nm infrared central illumination. Resolving power of the optics on the fundus is 250 line pairs per millimeter, with an imaging field-of-view of 4 deg × 4 deg. The rtx1 retinal camera illuminates the back of the subject's eye with a low-power beam of infrared light. A CCD camera within the instrument detects the optical image of the retina, which is then displayed on a computer screen. After adjusting the focusing depth and image localization in the retina, the user captures images that are stored in a database. The rtx1 retinal camera has 2 systems: a wavefront aberration correction system, and a lighting imaging system. Throughout the imaging process, wavefront aberrations are measured by Hartman-Shack (H-S) wavefront sensors, and a deformable mirror is used as a wave front corrector. After the wave front aberration of the human eye has been corrected by the adaptive optics system under the control of a computer, a flash lamp is triggered to illuminate the retina, and then high resolution images are captured by a CCD camera.

### 2.3 Acquiring fundus images

**Figure 2:**
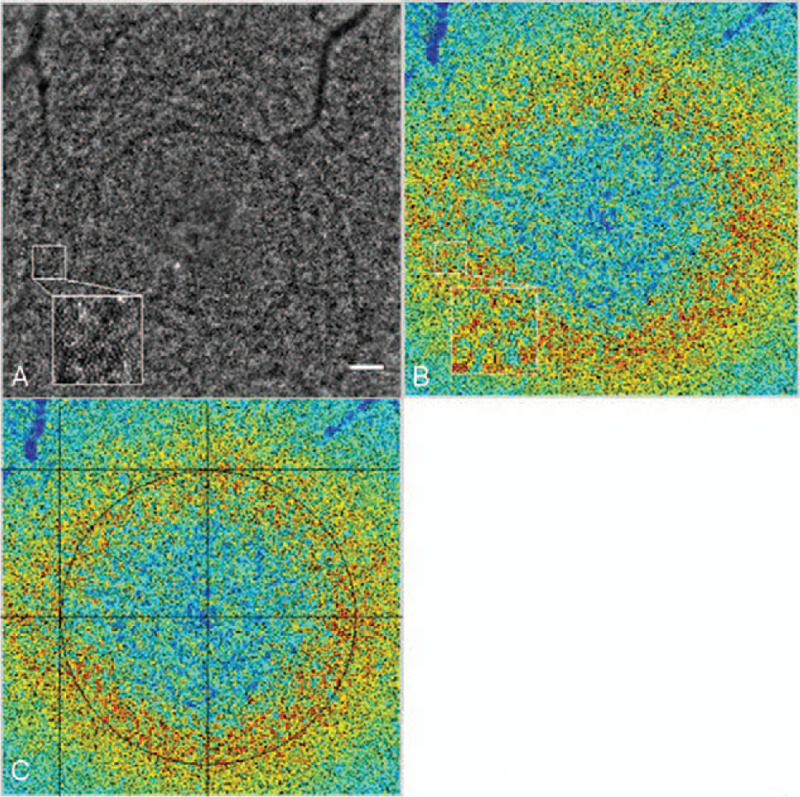
(A–C) The AO image of a right eye from a subject with BCVA 20/12.5. Images were processed with the analysis provided by AO detect 0.1 software. Bar calibration: 100 um. (A) The cone density from a subject with BCVA 20/12.5 presented as a color map. (B) A cone density map to locate the coordinates of the fovea. (C).

The subject was seated in front of the AO fundus camera within a comfortable chinrest, and then asked to fixate the center of its internal yellow cross. While looking at the live undilated pupil image, the investigator can position the green cross at the center of the 4 corneal-reflected retinal images. Then, the investigator adjusts the fixation target position and focuses the depth to obtain the best possible image, while maintaining the green cross aligned with the reflected images in the pupil image. Once the AO value displayed at the top of the live retinal image is stable, the patient to asked to blink twice, wait for 2 seconds and hold the eye steadily, and then an image is acquired. At the center of the fovea, using a CCD camera, a retinal image (Fig. 2A) made up of 40 individual frames was acquired in 4 seconds. Images of unacceptable quality were excluded from the study, which was less than 5%.

### 2.4 Calculation of cone parameters

The images obtained were processed by the software, which was supported by the manufacturer (AO detect v0.1 and AO detect v2.0). First, a cone density map (Fig. 2B) was obtained by the software (AO detect v0.1). Second, the cone density map showed a circle comprised of cones, which was used to locate the coordinates of the circle center, namely the fovea (Fig. 2C). Inter-investigator reproducibility was assessed, and the results of the cone parameters were compared by 2 masked investigators. The length and width of the screenshot were each 380 mm, and the center coordinates of the screenshot were x = A, y = B. The center coordinates of the retinal image were X_0_ = 1500∗ A/380–750, Y_0_ = 750–1500∗ B/380. The coordinates of 8 different retinal locations were calculated for 8 directions: temporal (X_1_ = X_0_–1500/4, Y_1_ = Y_0_), superior temporal (X_2_ = X_0_–1500/4∗cos45°, Y_2_ = Y_0_+1500/4∗sin45°), superior (X_3_ = X_0,_ Y_3_ = Y_0_+1500/4), superior nasal (X_4_ = X_0_+1500/4∗cos45°, Y_4_ = Y_0_+1500/4∗sin45°), nasal (X_5_ = X_0_+1500/4, Y_5_ = Y_0_), and inferior nasal (X_6_ = X_0_+1500/4∗cos45°, Y_6_ = Y_0_–1500/4∗sin45°), inferior (X_7_ = X_0_, Y_7_ = Y_0_–1500/4), and inferior temporal (X_8_ = X_0_–1500/4∗cos45°, Y_8_ = Y_0_–1500/4∗sin45°). Subsequently, these 8 80 × 80 μm areas on the retina at 1.5° of eccentricity from the fovea were sampled using the software (AO detect v2.0). The sampling areas were shifted slightly (5°) along a concentric circle of the fovea, when vessels were obvious and occlusive in those areas, which was rare. The density and spacing of the cones for the 8 sampling areas were averaged. The effects of ocular AL were adjusted for the retinal magnification factor (RMF). To transfer the unit from a line scale to an angle, the AO system was used with the formula RMF to convert the cone parameters.^[19]^ Assuming the AL as x, the RMF was calculated using the following equation: RMF = 0.01306∗(x −1.82). RMF represents the transformation from degrees to mm across the retina.

### 2.5 Statistical analysis

Data are presented as the mean ± 1 standard deviation. Statistical analysis was performed using an SPSS 23 software program (SPSS, Chicago, IL) and MedCalc statistical software (version 16.2.0). Differences between the 2 test groups were estimated using the Student *t* test when the data were expressed as a normal distribution, or using the Mann–Whitney *U* test when the data were a non-normal distribution. Gender between the study groups was evaluated by a Chi-Squared test. A binary logistic regression was applied for the analysis between visual resolution and factors that were ocular biological features and cone parameters. The univariate analysis and multivariate stepwise analysis using binary logistic regression were also performed. Statistical significance was set at 0.05 for all tests. The range of agreement between the 2 investigators was assessed using the Bland–Altman method.^[20,21]^

## 3 Results

Seventeen eyes of 10 subjects were assessed in group 1 (BCVA ≥ 20/12.5), and 16 eyes of 10 subjects were assessed in group 2 (BCVA = 20/16). Age (*P* = .755) and gender (*P* = .226) were not significantly different between the 2 groups. The mean ± SD AL was 23.93 ± 0.91 mm and 25.27 ± 0.96 mm in group 1 and group 2, respectively, being lower in group 2 (*P* < .001). The mean spherical equivalent of group 1 and group 2 was −0.25 D and −2.94 D (*P* = .022), respectively. All characteristics of the study subjects are presented in Table 1. The results of the Bland–Altman analysis demonstrated that the average difference between investigators was 123.3 cells/mm^2^ for cone density (Table 4A), 10.2 cells/deg^2^ for angular cone density (Table 4B), 0.02 μm for cone spacing (Table 4C), 0.004 arc minutes for angular cone spacing (Table 4D), and 0.5 for cone number (Table 4E), all being very small.

**Table 1:** Characteristic between the 2 groups.

Parameter	Group 1 (N = 17)	Group 2 (N = 16)	*P*
Age (yr)	24 (22,26)	25 (23,25)	.755
Gender			
Male	5 (29.4%)	8 (50.0%)	.226
Female	12 (70.6%)	8 (50.0%)	
Sp (D)	−0.25 (−1.25, 0.13)	−2.63 (−4.00, 0.19)	.020
Cy (D)	0.00 (−0.25, 0.00)	−0.38 (−1.00, 0.00)	.066
SE (D)	−0.25 (−1.25, 0.00)	−2.94 (−4.09, −0.47)	.022
AL (mm)	23.93 ± 0.91	25.27 ± 0.96	<.001
CCT (μm)	543.88 ± 33.43	532.06 ± 23.78	.253
AD (mm)	3.06 ± 0.36	3.17 ± 0.26	.307
LT (mm)	3.67 ± 0.34	3.58 ± 0.16	.336
K1 (D)	42.54 ± 1.67	42.02 ± 1.07	.296
K2 (D)	43.28 ± 1.75	42.71 ± 1.09	.274
AST (D)	0.69 (0.49, 0.76)	0.68 (0.45, 0.82)	.871

The mean ± SD cone density at 1.5° retinal eccentricity in group 1 was 29,570.96 ± 2489.94 cells/mm^2^ and 22,963.59 ± 2987.92 cells/mm^2^ (*P* < .001) in group 2. According to the RMF that converted scale lines to angles, the mean ± SD of angular cone density at 1.5° retinal eccentricity of group 1 was 2454.98 ± 98.65 cells/deg^2^, and larger than that of group 2 at 2142.62 ± 203.17 cells/deg^2^ (*P* < .001). In the study subjects, the mean ± SD cone spacing at 1.5^°^ in group 1 and group 2 was 6.45 ± 0.28 μm and 7.36 ± 0.50 μm, respectively (*P* < .001). The effect of AL was adjusted with the mean angular cone spacing at 1.5^°^, and it was smaller in group 1 (*P < *.001), as seen in Table 2.

**Table 2:** Cone density and cone spacing of the 2 groups.

Parameter	Group 1 (N = 17)	Group 2 (N = 16)	*P*
Cone number	112.35 ± 4.47	98.00 ± 9.29	<.001
Cone density (cell/mm^2^)	29,570.96 ± 2489.94	22,963.59 ± 2987.92	<.001
Angular cone density (cell/deg^2^)^∗^	2454.98 ± 98.65	2142.62 ± 203.17	<.001
Cone spacing (μm)	6.45 ± 0.28	7.36 ± 0.50	<.001
Angular cone spacing (arcmin)^∗^	1.34 ± 0.03	1.44 ± 0.07	<.001

The results of the binary logistic regression analyses for exploring the relationship between the variance of visual acuity and optical biometry are presented in Table 3. In the univariate logistic regression model, cone number (odds ratio [OR], 1.37; *P* = .004), cone density (OR, 2.72; *P* = .003), angular cone density (OR, 4.06; p = .004), cone spacing (OR, 0.53; *P* = .003), angular cone spacing (OR, 0.62; *P* = .004), spherical power (Sp) (OR, 1.88; *P* = .014), cylinder power (OR, 10.01; *P* = .038), SE (D) (OR, 1.83; *P* = .012), and AL (OR, 0.21; *P* = .005) were significantly related to BCVA ≥ 20/12.5. Age, gender, central corneal thickness (CCT), AD, lens thickness, flat meridian, steep meridian, and astigmatism were not significantly related to BCVA ≥ 20/12.5, as presented in Table 3. Cone number, angular cone density, and angular cone spacing were separately analyzed using a stepwise analysis with other optical biometry features. Results of this stepwise analysis revealed that cone number (OR, 1.39; *P* = .005), angular cone density (OR, 4.48; *P* = .005), and angular cone spacing (OR, 0.60; *P* = .007) remained significantly related to BCVA ≥ 20/12.5.

**Table 3:** Univariate binary logistic regression in different groups.

	Univariate model 1			Univariate model 2		
	Odds	95% Confidence	*P*	Odds	95% Confidence	*P*
Factors	ratio	interval	value	ratio	interval	value
Age (yr)	0.90	0.66–1.22	0.494			
Gender	2.40	0.57–10.04	0.231			
Sp (D)	1.88	1.14–3.10	0.014	1.82	1.09–3.03	.022
Cy (D)	10.01	1.13–88.28	0.038	9.17	0.82–102.38	.072
SE (D)	1.83	1.14–2.93	0.012	1.78	1.10–2.90	.020
AL (mm)	0.21	0.07–0.62	0.005	0.16	0.04–0.64	.009
CCT (μm)	1.02	0.99–1.04	0.252	1.02	0.99–1.04	.241
AD (mm)	0.28	0.03–3.05	0.299	0.41	0.03–5.44	.501
LT (mm)	4.40	0.25–77.77	0.312	3.54	0.17–75.46	.419
K1 (D)	1.32	0.79–2.22	0.291	1.24	0.72–2.16	.439
K2 (D)	1.33	0.80–2.19	0.269	1.25	0.74–2.11	.416
AST (D)	1.39	0.23–8.38	0.719	1.34	0.20–8.88	.762
Cone number	1.37	1.11–1.69	0.004	1.37	1.10–1.69	.004
Cone density (cell/mm^2^)	2.72	1.60–4.61	0.003	2.72	1.32–6.84	.009
Angular cone density (cell/deg^2^)	4.06	1.52–10.80	0.004	4.06	1.52–10.80	.004
Cone spacing (μm)	0.53	0.35–0.80	0.003	0.41	0.21–0.81	.010
Angular cone spacing (arcmin)	0.62	0.45–0.86	0.004	0.62	0.45–0.86	.005

**Table 4:**
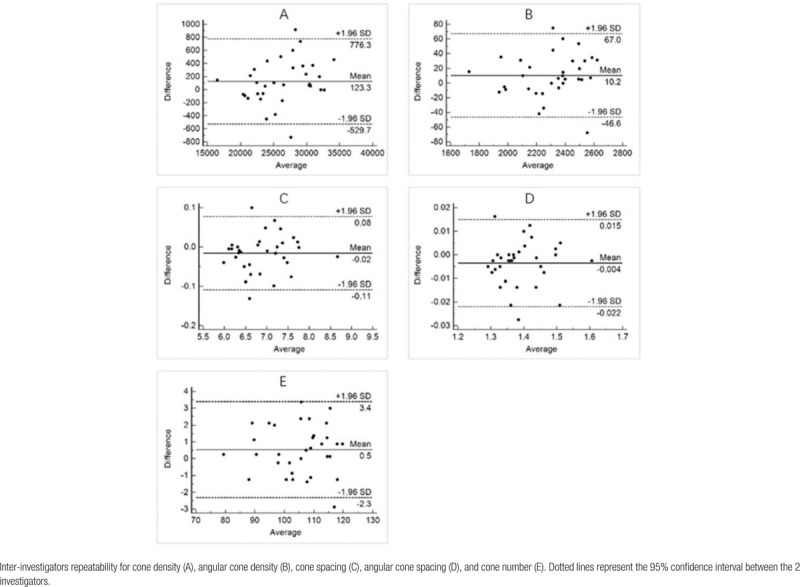
Bland–Altman diagrams.

## 4 Discussion and conclusions

In the present study, the AO fundus camera was used to measure the cone parameters of the retina. The axial lengths between the 2 groups (BCVA 20/12.5 or better vs BCVA 20/16) were significantly different (*P < *.001). However, the mean angular cone density at the parafovea was larger, and the mean angular cone spacing at the parafovea was smaller, for the group with BCVA of 20/12.5 than the group with BCVA of 20/16, with the cone parameters converted using a RMF. In addition, less refractive error and shorter axial lengths were significantly associated with BCVA 20/12.5. In a previous study, AL and SE were significantly correlated with reduced visual resolution in high myopia without ocular pathology.^[22]^ And in an earlier study with a larger sample size, AL and SE increased with higher prevalence of visual Impairment due to myopia.^[23]^ In the present study, only subjects with normal visual acuity were investigated, and yet significant differences between visual acuity, AL, Sp, and SE were found between the 2 groups. However, according to the results of the stepwise regression, smaller angular cone spacing and greater angular cone density at the parafovea were still strongly associated with BCVA of 20/12.5 or better, and there was no significant difference in this analysis with AL, Sp, and SE. To our knowledge, this is the first study to explore the relationship between visual resolution and selected cone parameters at the parafovea in eyes with different BCVA levels, but with all being normal in value.

Data for the cone density (cones/mm^2^ [mean ± SD]) at 1.5^°^ from the fovea were 29,570.96 ± 2489.94 and 22,963.59 ± 2987.92 for group 1 and 2, respectively. The cones numbers are likely underestimated because cones were counted using an automated algorithm, however, since we evaluated cone parameters between the 2 groups with the same method, this would have the same impact on the comparative group results. These present results are in agreement with previous AO studies that assessed the cone parameters in healthy eyes with normal visual acuity levels. For example, Dabir et al^[17]^ calculated the mean cone density of subjects with BCVA of 20/20 or better at 2^°^ (2^°^eccentricity = 25350/mm^2^), and their findings correlated closely with the current ones. Muthiah et al^[13]^ also reported that the photoreceptor densities were 26,500 (manual) cones/mm^2^ and 24,200 (automated) cones/mm^2^ at a retinal eccentricity of 2^°^, which were also similar to the present study, and their subjects also had a BCVA of 20/20 or better. On the other hand, the cone parameters at 1.5° from the fovea in the present study did not agree with those (approximately 40,000 cones/mm^2^) of Sawides.^[24]^ There are 2 possible reasons for this discrepancy. First, the assessment methods were different. Second, the analyses were different. Further studies in this area are therefore warranted.

Prior to the development of the AO fundus camera, the data of cone photoreceptors were usually obtained histologically. Cone density data for eyes with BCVA of 20/16 are in a reasonable range with the data published by Jonas et al^[1]^ acquired by histological count. However, Curcio et al^[2]^ analyzed cone densities postmortem. They reported that cone density at 2^°^ of retinal eccentricity was approximately 40,000 cells/mm^2^. Most subjects from other similar studies had cone densities less than those recorded here. This could be explained by possible shrinking of the postmortem eyes, and also by individual differences in the eyes, especially with relatively small sample sizes (e.g., <50 eyes). Previously, Chui et al^[25]^ demonstrated that the mean cone density of healthy subjects with BCVA of 20/20 or better was 30,000 cells/mm^2^, which was approximately equal to the cone density at 1.5^°^ in the present group with BCVA 20/12.5 or better. Perhaps some subjects having BCVA 20/12.5 or better were recruited in that study. Cone spacing at 1.5^°^ from the fovea in the group with BCVA 20/16 was 7.36 ± 0.50 μm, greater than that in the group with BCVA 20/12.5 or better (6.45 ± 0.28 μm). The data of Muthiah et al^[13]^ showed that the mean spacing of cones was 6.8 μm at 2^°^ from the fovea, which is similar to that found in the current study. In addition, the present results are in agreement with a previous study by Dabir et al^[17]^ using an AO retinal camera, in which the researchers reported that the mean cone spacing was 6.9 μm at 2^°^ eccentricity, with a sample size of 25 healthy subjects.

The current investigation provides several new viewpoints. Cone spacing at the parafovea was highly correlated with each of the 2 different normal BCVA levels. The visual quality of the human eyes is affected by ocular aberrations.^[26]^ The theoretical limit of visual acuity is BCVA of better than 20/12.5, obtained following the custom correction of an individual's optical aberrations.^[27]^ However, the present study did not include subjects within the theoretical limits of visual acuity, cone spacing may be smaller at the theoretical limits of visual acuity. More studies of the relationship between the theoretical limits of visual acuity and the various cone parameters may help us to understand these and related findings better.

The concept of high resolution vision function is still in its infancy. For example, Zhou et al^[28]^ reported that human visual acuity corrected for high-order aberrations (HOAs) could be further improved by perceptual learning involving brain plasticity. However, Park et al^[29]^ reported thinning of the ganglion cell layer and inner plexiform layer in amblyopic eyes as compared to their fellow normal eyes. They speculated that these anatomical changes may contribute to the visual acuity differences. Interestingly, the present study demonstrated a close relationship between visual resolution and cone parameters at the parafovea, and this may provide a new direction for the study of normal and abnormal vision function. In addition, we will be able to analyse healthy eyes using AO fundus camera, and use them to build the normative databases. This can be used in clinical practice to help clinicians identify the ophthalmic disease using AO.

Some the limitations of the present study include a relatively small sample size, and the data being confined to only 1.5^°^ of retinal eccentricity and thus not representative of the fovea due to limitations of the rtx1, and automated cone analysis that underestimated the actual number. In addition, this study subjects without any refractive error were not included in this study. However, the effect of ocular AL was eliminated due to the retinal magnification factor.

In summary, this is the first report showing that mean cone density at the parafovea was greater, and mean cone spacing was smaller, in a group of BCVA of 20/12.5 or better as compared to those with BCVA of 20/16. The cone parameters of the parafovea were strongly associated with visual resolution rather than with either AL or SE, and furthermore are in reasonable agreement with the earlier histological data and other AO studies. Understanding cone spacing and density distribution for different BCVA levels may be beneficial for gaining insight into different clinical conditions, ranging from supernormal vison to amblyopia. Further studies are required to focus on elucidating the relationship between BCVA and cone parameters at the fovea in both normal and abnormal clinical populations.

## Author contributions

**Conceptualization:** Jingwei Zheng.

**Data curation:** Kun Zhou, Sigeng Lin.

**Investigation:** Yuanbo Liang.

**Validation:** Huanhuan Cheng, Huilu Jiang, Jingwei Zheng.

**Writing – original draft:** Huanhuan Cheng.

**Writing – review & editing:** Kenneth J. Ciuffreda, Xinping Yu, Balamurali Vasudevan, Yuanbo Liang.
